# Multilayer Perceptron-Based Error Compensation for Automatic On-the-Fly Camera Orientation Estimation Using a Single Vanishing Point from Road Lane

**DOI:** 10.3390/s24031039

**Published:** 2024-02-05

**Authors:** Xingyou Li, Hyoungrae Kim, Vijay Kakani, Hakil Kim

**Affiliations:** 1 Electrical and Computer Engineering, Inha University, 100 Inha-ro, Michuhol-gu, Incheon 22212, Republic of Korea; 2 Future Vehicle Engineering, Inha University, 100 Inha-ro, Michuhol-gu, Incheon 22212, Republic of Korea; 3 Integrated System Engineering, Inha University, 100 Inha-ro, Nam-gu, Incheon 22212, Republic of Korea

**Keywords:** autonomous vehicles, camera orientation estimation, vanishing point, camera extrinsic parameters

## Abstract

This study introduces a multilayer perceptron (MLP) error compensation method for real-time camera orientation estimation, leveraging a single vanishing point and road lane lines within a steady-state framework. The research emphasizes cameras with a roll angle of 0°, predominant in autonomous vehicle contexts. The methodology estimates pitch and yaw angles using a single image and integrates two Kalman filter models with inputs from image points (u, v) and derived angles (pitch, yaw). Performance metrics, including avgE, minE, maxE, ssE, and Stdev, were utilized, testing the system in both simulator and real-vehicle environments. The outcomes indicate that our method notably enhances the accuracy of camera orientation estimations, consistently outpacing competing techniques across varied scenarios. This potency of the method is evident in its adaptability and precision, holding promise for advanced vehicle systems and real-world applications.

## 1. Introduction

### 1.1. Background

With the advancements in vision sensor technology, the utilization of vision sensors in advanced driving-assistant systems (ADAS) has become prevalent. The organic incorporation of vision-based sensing technologies such as lane-keeping systems (LKS) and forward collision warnings (FCW) into self-driving scenarios has begun to improve. Most functions are based on vision systems, such as cameras, to obtain the visual context of human perception. However, this pipeline approach of streamlining vision-based inputs to obtain plausible outcomes is common in the ADAS-based industry for accomplishing multiple tasks, such as object detection and segmentation. This is continuously widening the sensing capabilities of self-driving systems [1]. Lane detection is a basic task of an ADAS to identify lane boundaries and extend these to attain LKS, lane departure warning (LDW), and vanishing point detection. Commercial products such as open pilots can be considered successful cases of combining object detection and semantic segmentation into a product with vertical coverage from sensing to control. The simulator also contributes significantly to the regulation and simulation of control systems [2].

Camera calibration is also a sub-problem in computer vision. It mainly analyzes known scenes from a sensor from various perspectives and uses these to calibrate the sensor. For example, in the FCW, a practical calculation of the distance between the vehicle and the object ensures a safe distance for the vehicle. This can be affected by the terrain and weather conditions. Single-camera distance estimation assumes that the object and vehicle are on the same plane and projects a 3D object onto a 2D image using calibrated intrinsic and extrinsic parameters of the camera [3]. Because the intrinsic parameters would have been established at the time of production, these do not vary under normal conditions. Meanwhile, the extrinsic parameters represent the relationship between the target coordinate system and the camera coordinate system. The extrinsic parameters are generally updated according to the motion of the vehicle.

The accurate camera model and the relationship between it and the world are helpful for sensing 3D worlds such as object-based distance estimation [4]. To describe the camera model as intrinsic parameters including the focal length and principal point, and to describe the relationship between the camera model and the world, always use rigid body transformation, also known as extrinsic parameters. In a self-driving vehicle environment, the camera intrinsic parameters are always stable with temperature changes and vehicle shaking, and the camera extrinsic parameters always change when the camera changes. However, conventional camera calibration methods require a calibration target (typically a 2D plane of known size and shape, such as a checkerboard) to determine the camera parameters, thus constituting off-line calibration. As for online calibration, it has a long processing time or needs more images as a reference for better performance [5,6]. Therefore, accurate on-the-fly camera calibration is needed on the vehicle for several applications.

### 1.2. Purpose of Study

**Figure 1:**
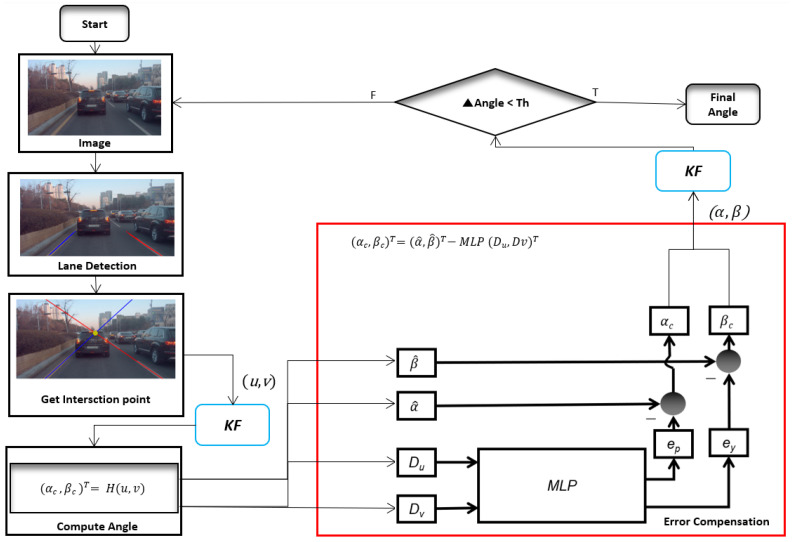
The framework for automatic on-the-fly camera orientation estimation system.

**Figure 2:**
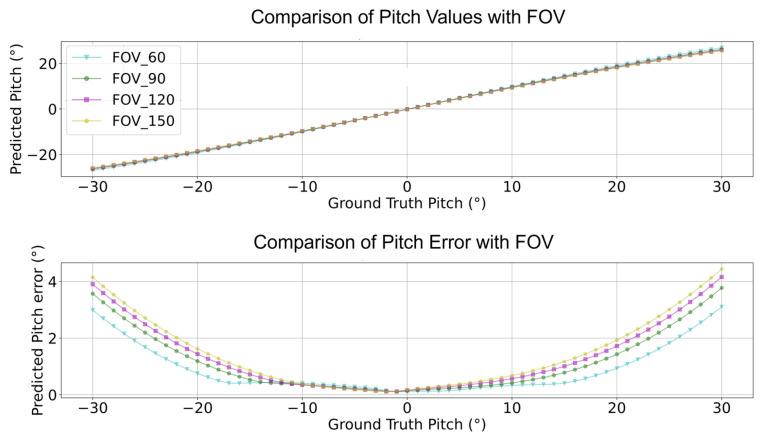
$f\left(u,v\right),e_{p}$ for pitch performance with different camera FOVs.

**Figure 3:**
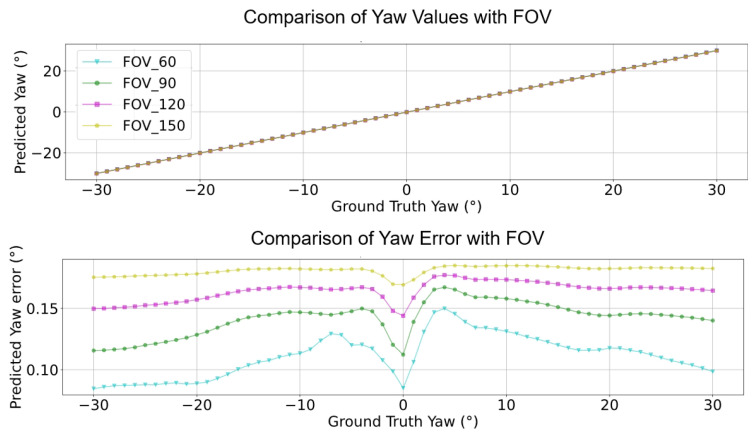
$f\left(u,v\right),e_{p}$ for yaw performance with different camera FOVs.

The proposed research was conducted in relevance to the error compensation method for on-the-fly camera orientation estimation such as multilayer perceptron (MLP). The overall study analysis is depicted in Figure 1. Often, traditional online camera orientation methods accumulate errors in the camera orientation estimation and do not consider error compensation factors, as shown in Figure 2 and Figure 3, which was rectified in the proposed method. Accordingly, the contributions of this study are as follows:

- A working pipeline for the MLP-based adaptive error correction automatic on-the-fly camera orientation estimation algorithm with a single VP from a road lane is proposed, along with relevant quantitative analysis.
- A stable camera on-the-fly orientation estimation variant is proposed. It uses a Kalman filter that can estimate the angular pitch and yaw concerning the road lane.
- The residual error of using VP to estimate the camera orientation is compensated for, and several related compensation modules are compared.

The remainder of the paper is organized as follows. The related work is presented in Section 2. This is followed by the proposed method, including the rotation estimation, system of estimation, and evaluation metrics in Section 3. The experimental results are presented in Section 4. The performance of the proposed system and ablation study are discussed in Section 5. Section 6 discusses the limitations of the present study and future work. Section 7 concludes this study.

## 2. Related Work

Fundamental camera calibration can be categorized into two types: intrinsic (focal length, camera center, and distortions) [7] and extrinsic, which involves mapping rotations and translations from the camera to the world coordinates [8,9] or other sensor coordinates [10,11]. There are two methods involved in on-the-fly the camera orientation estimation for camera extrinsic calibration using target-less methods. One is to determine the area of the object to estimate the yaw and pitch in [12]. The other is to identify the vanishing point (VP) to estimate the orientations. Hold et al. used periodic dashed lanes to estimate the initial extrinsic parameters [13]. Paula et al. developed a model to estimate using the VP [14]. Lee et al. estimated the pitch and yaw using a new method in conjunction with VPs [15]. Jang et al. estimated the three angles using three VPs [16]. Guo et al. also developed indoor applications using VPs [17]. This study focuses on the orientation between the camera and world coordinates. To obtain rotations and translations for online camera calibration, targets such as registered objects [18,19], road marker lanes [20], and objects with apparent appearances [21] are commonly used. However, these methods cannot cover the case regarding varying extrinsic parameters. The authors of [14] proposed another model to estimate the pitch, yaw, and height for the target-less calibration methods. The generated case was used for extrinsic parameter estimation. The authors of [15] proposed an online extrinsic camera calibration method that estimates pitch, yaw, roll angles, and camera height from road surface observations by using a combination of VP estimation and lane width prior. The authors of [17] used a more detailed version to estimate the absolute orientation angle. It included the pitch and yaw. They used a method similar to that of the authors of [15] and analyzed the failure cases. Although they utilized specific applications considering auto-calibration, their approach had significant errors. These methods cannot prevent projection errors even with an appropriate VP. The authors of [16] proposed an online extrinsic calibration approach for estimating camera orientation using motion vectors and line structures in an urban driving environment from three VPs using 3-line RANSAC [22] on a Gaussian sphere. However, the method required more reference lines in the image, and the algorithm was easy to use to obtain failed results. To solve this problem, the concept of the reprojection root-mean-squared error [23] for pixels was used. It is a metric used to express the calibration error. This method is a camera-setup-independent error metric used to measure the performance of the calibration algorithm (our model) while omitting extrinsic influences.

For the calibration system, the authors of [15,16] proposed stabilization systems using an extended Kalman filter (EKF) to solve the nonlinear system. However, Ref. [16] needs more reference lines to obtain several sets of candidate VPs from 3-line RANSAC on a Gaussian sphere. Therefore, a considerably long time is required to complete the process. The VPs also fail because the selected lines intersect at infinity. Study [15] has a residual error based on the distance from VP and the center point(CP) of the image.

This study proposes an MLP-based error compensation method for automatic on-the-fly camera orientation estimation with a single VP from the road lane and steady-state systems. The method considers the commonly available scenes around a self-driving vehicle. It estimates the pitch and yaw with the correction part using an image.

## 3. Proposed Method

### 3.1. Camera Orientation Estimation

**Figure 4:**
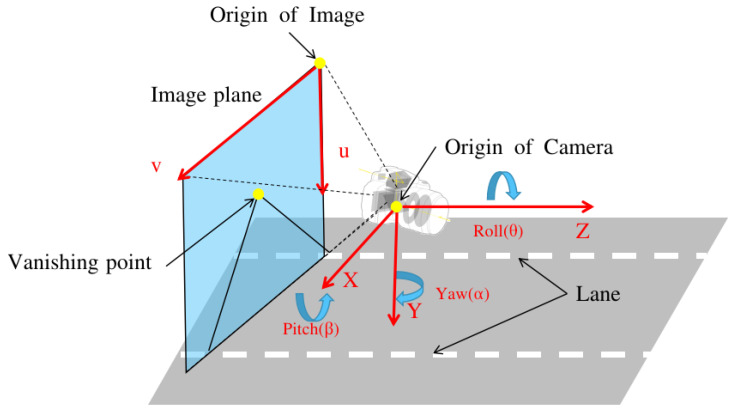
Camera orientation to road plane and coordinates definition for each coordinate system.

It is established that parallel lines do not intersect in the world coordinate system. Thus, we can conclude that the VPs are located at an infinite distance from each other. If we consider the forward direction of a vehicle parallel to the lane lines, we can determine that the lane lines intersect at z=∞. The road reference frame is denoted by the point (*X, Y, Z*), where *Z* corresponds to the forward axis of the vehicle. It should be noted that the road reference frame is fixed to the vehicle, as shown in Figure 4. Assumptions regarding the alignment of the vehicle with the lane and lane straightness are crucial. This is because the VP, the intersection point of the lane lines in the image, can provide information regarding camera mounting. Specifically, it reveals the orientation of the camera relative to a vehicle. However, if this assumption is not satisfied, the VP would only provide information regarding the orientation of the vehicle to the lane lines and not that regarding the camera orientation.

The camera projection equation with $P_{i}$ as a point in the image coordinates and $P_{r}$ as a point in the real-world coordinates is as follows:

(1) $$sP_{i}=K\left[R|T\right]P_{r}$$

(2) $$P_{i}={\left(u,v,1\right)}^{T}$$

(3) $$P_{r}={\left(X,Y,Z,1\right)}^{T}$$

where *K* refers to the intrinsic parameter, *R* to the rotation matrix, and *T* to the translation matrix. Because this is an equation in homogeneous coordinates, we can multiply both sides by a scalar value s and absorb this value into *Z* on the left-hand side of the equation:

(4) $$s\left(\begin{array}{c} u\\ v\\ 1 \end{array}\right)=K*R|T\left(\begin{array}{c} X/Z\\ Y/Z\\ 1\\ 1/Z \end{array}\right)$$

The *z*-axis value of the VP in the world coordinates is infinite. Thus, (4) becomes

(5) $$s\left(\begin{array}{c} u\\ v\\ 1 \end{array}\right)=K*R|T\left(\begin{array}{c} 0\\ 0\\ 1\\ 0 \end{array}\right)$$

The rotation matrix is composed of pitch, yaw, and roll angles. During the setup, the camera is mounted on the windshield, which is parallel to the road trajectory, and under such circumstances, the roll angle defaults to zero. Thereby, the rotation matrix is as follows:

(6) $$R=\left(\begin{array}{ccc} r_{11} & r_{21} & r_{31}\\ r_{12} & r_{22} & r_{32}\\ r_{13} & r_{23} & r_{33} \end{array}\right)$$

As previously assumed for the roll degree, the rotation matrix represents the yaw and pitch as follows:

(7) $$R=\left(\begin{array}{ccc} cos(\alpha ) & sin(\alpha )sin(\beta ) & sin(\alpha )cos(\beta )\\ 0 & cos(\beta ) & -sin(\beta )\\ -sin(\alpha ) & cos(\alpha )sin(\beta ) & cos(\alpha )cos(\beta ) \end{array}\right)$$

As shown in (5), when (6) is substituted into (5), only the third column of information remains. Therefore, the following formula can be used for the two angles:

(8) $$\left(\begin{array}{c} \alpha_{c}\\ \beta_{c} \end{array}\right)=\left(\begin{array}{c} atan2(r_{31},r_{33})\\ -arcsin\left(r_{32}\right) \end{array}\right)$$

where $\alpha_{c}$ is yaw and $\beta_{c}$ is pitch radian angle.

Accordingly, the reprojection errors can be estimated using the calculated VPs, pitch, and yaw information. Furthermore, the reprojection error ($e_{p},e_{y}$) distribution of angles with respect to various camera FOVs can be obtained (see Figure 2 and Figure 3). The function that obtains the reprojection error is shown as:

(9) $$\left(\begin{array}{c} e_{p}\\ e_{y} \end{array}\right)=\sum_{i=1}^{n}\left(f\left(u,v\right)-\left(\alpha_{c},\beta_{c}\right)\right)$$

where the function f(u,v) is defined as follows:

(10) $${\left(\hat{\alpha },\hat{\beta }\right)}^{T}=f{\left(u,v\right)}^{T}$$

where $\hat{\alpha }$,$\hat{\beta }$ represent the estimated yaw and pitch angle.

### 3.2. Error Compensation of Orientation Estimation

As shown in Equation (9), the error residuals are determined by the camera pose angles as depicted in Equation (8), referring to the position of the VP. The pitch angle $\beta_{c}$ and yaw angle $\alpha_{c}$ are mathematically represented by the *sin*() and *tan*() elements as shown in Equation (8); accordingly, the residuals used in the process of estimating the reprojection error as shown in Equation (8) use this pitch and yaw information. During this, the residual pitch and yaw angles are estimated using the distance from the center of the picture to the edge, the residual pitch is approximated by a quadratic function as shown in Figure 2, and the residual yaw is approximated as an even function at the zero point as shown in Figure 3.

**Figure 5:**
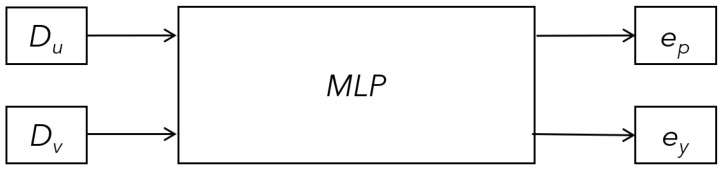
The MLP model to estimate the error with the VP: The two inputs are the distance from the CP of the image to the VP, whose description as $D_{u}$ and $D_{v}$ on the image coordinates. The two outputs are the pitch and yaw error for error compensation, whose description is $e_{p}$ and $e_{y}$ on the image coordinates.

MLP [24] is a conventional method for determining nonlinear coefficients. Considering that the complexity of the nonlinear relationship is moderate and that the correlation is quasi-linear, this study used a shallow network with a rectified linear unit (ReLU) activation function (see Figure 5). The MLP model is for the VP to estimate the pitch and yaw errors. The two inputs are the distances from the VP to the CP. These are described as $D_{u}$ and $D_{v}$ in the image coordinates. The two outputs of the model are the pitch $e_{p}$ and yaw $e_{y}$ errors for error compensation and there by Equation (9) can be extended to Equation (13) using image coordinates of the distances from the VP to the CP whuch are $D_{u}$ and $D_{v}$.

After the MLP model, the error compensation part would function as follows:

(11) $$H\left(D_{u},D_{v}\right)={\left(\hat{\alpha },\hat{\beta }\right)}^{T}-MLP{\left(D_{u},D_{v}\right)}^{T}$$

(12) $$\left(D_{u},D_{v}\right)=\vec{vp}-\vec{cp}$$

(13) $$\left(\begin{array}{c} e_{p}\\ e_{y} \end{array}\right)=MLP{\left(D_{u},D_{v}\right)}^{T}$$

### 3.3. Multilayer Perceptron

In this section, we utilize an MLP model as the core component for the error compensation of the angle estimation from the VP. The MLP architecture comprises an input layer, at least one hidden layer, and an output layer. This neural network design aims to generate a specific flow of information between the layers to learn complex representations of the input data.

**Figure 6:**
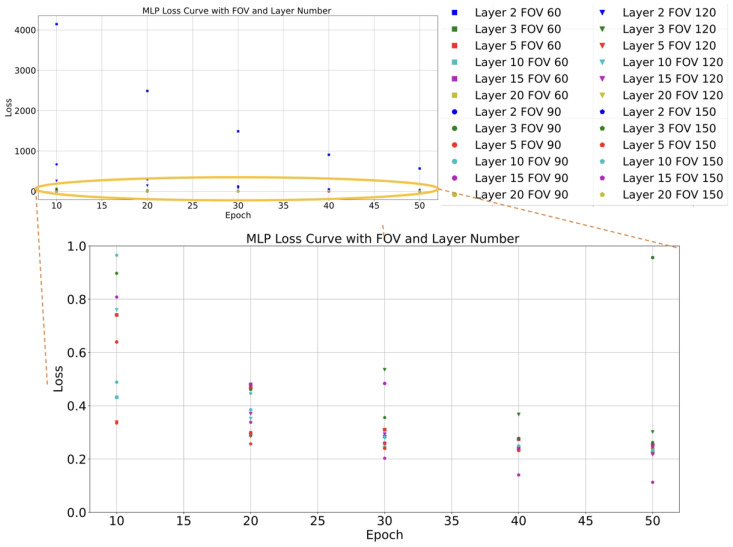
Loss curves: The training data comprise the distance from VP to CP and the related error. The validation data comprise different FOV data. Networks are built by different layers.

**Figure 7:**
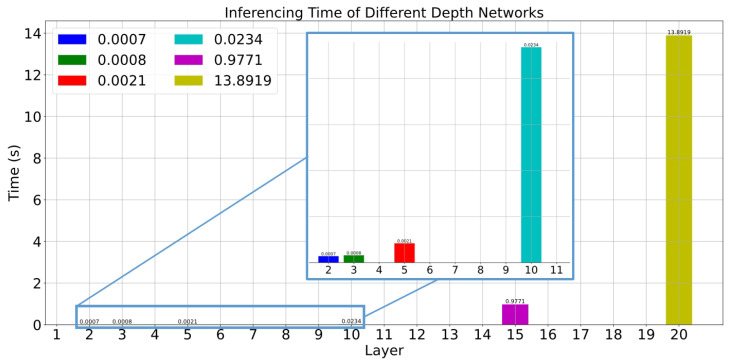
Average inferencing time for each network.

Figure 6 and Figure 7 show the performance of each network with different FOV data. The layers of all networks are progressive to the power of 2. For example, the first layer is $2^{5}$, and the second layer is $2^{6}$. Considering the loss and inferencing time, the network with 5 layers are best one to build the on-the-fly system. The first hidden layer receives two input features and maps these to a 64-dimensional feature space. The subsequent layers increase the feature space to 128 dimensions, gradually decrease it to 64 and 32 dimensions, and produce a 2-dimensional output. The layers are connected through linear transformations (weights and biases) and activation functions such as ReLU. The number of neurons in each layer enables the network to learn higher-level features and abstractions to fit the error distribution. And the loss almost always converges at the 50th epoch.

## 4. Experimental Result

### 4.1. Experimental Environment: Simulation and Real

This section presents the settings and scenarios used in this study. First, to ensure the authenticity of the algorithm verification environment while considering the accuracy and operability of the ground truth (GT) data, the current city simulator was selected (see Table 1). The table outlines the main features and functions of certain highly effective emulators, such as the image resolution, FOV, camera model, camera pose, map generation engine, and minimum hardware requirements. Among these, MORAI has relatively high accuracy. This map of South Korea was selected as the experimental simulator. An experiment was conducted on IONIQ5, which is used for self-driving research.

Table 1 shows the differences. Here, the platforms offer a range of image resolutions. Most of these support a maximum of 1920 × 1920 pixels. The FOV for these platforms also varies. A few provide a more comprehensive range of up to $180^{\circ }$ and $200^{\circ }$. The camera models utilized by these platforms can be ideal or physical. Specific platforms support both types. The rotation indicates the camera poses *R* and translation *T* values. These demonstrate the precision in the simulation environment.

**Figure 8:**
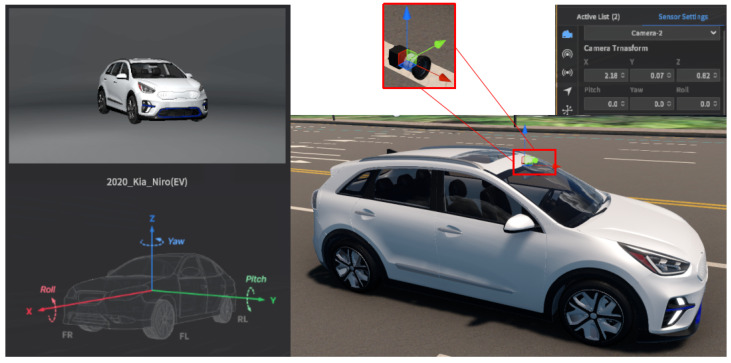
The settings of MORAI environment and camera.

From the survey of the simulators, the setting of the MORAI simulator was more suitable for this study. Moreover, it supports the city map of South Korea. The experimental setup of the emulator used in this study is shown in Figure 8. The map was that of Sangam-dong in Seoul, Republic of Korea. The vehicle model was the Kia Niro(EV) 2020 version. The camera model was the default one that supports undistorted image information with adjustable FOV and image resolution. The sensor position and orientation were based on the car coordinates.

**Figure 9:**
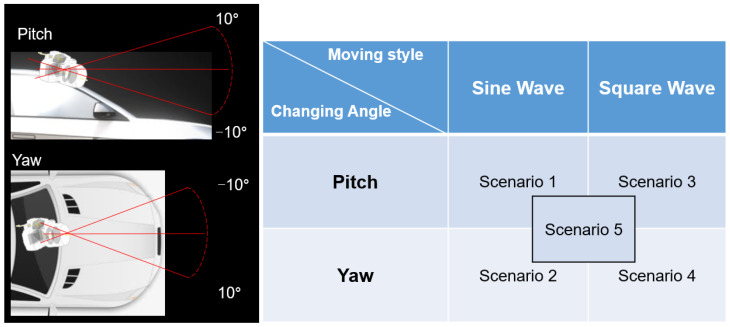
Scene settings based on transformed angles and changing angles.

To evaluate the stability of the pitch and yaw angle estimation and verify the performance of angle prediction, one angle was fixed, and the other was varied. The scenarios of the experiments are designed as follows in Figure 9:

- Scenario 1: For the algorithm to follow the pitch motion with a 0.1° variation, it modifies the pitch angle continuously while the yaw angle is fixed to $0^{\circ }$.
- Scenario 2: For the algorithm to follow the yaw motion with a 0.1° variation, the yaw angle is modified continuously while the pitch angle is fixed to $0^{\circ }$.
- Scenario 3: For the system to converge in certain frames to correct the pitch angle with a $10^{\circ }$ variation, the pitch angle is modified by $10^{\circ }$ in each frame while the yaw angle is fixed to $0^{\circ }$.
- Scenario 4: For the system to converge in certain frames to correct the yaw angle with a $10^{\circ }$ variation, the yaw angle is modified by $10^{\circ }$ in each frame while the pitch angle is fixed to $0^{\circ }$.
- Scenario 5: For the system to converge in certain frames to correct the angle with a $10^{\circ }$ variation, the pitch and yaw angle is modified by $1^{\circ }$ in each frame.

**Figure 10:**
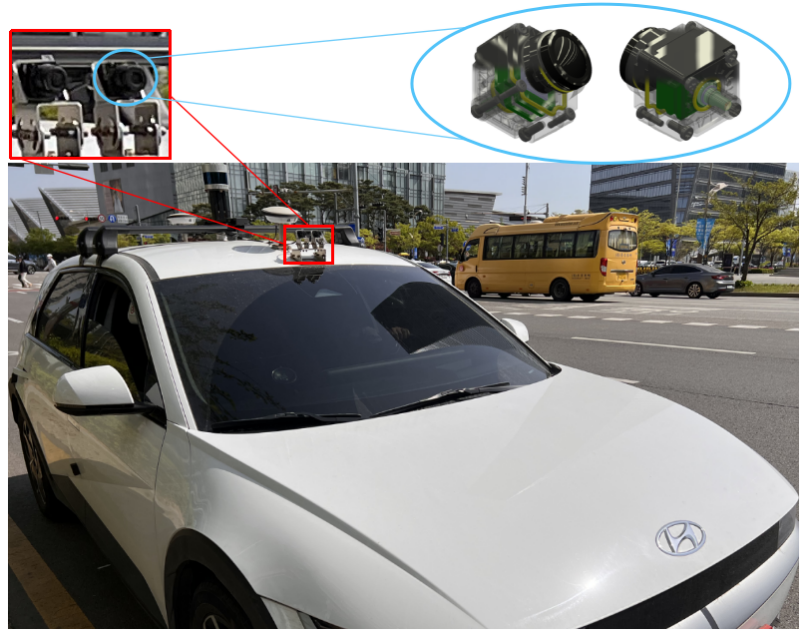
The settings of real vehicle and camera.

To better verify the algorithm in the real world, experiments related to real data were conducted on IONIQ5 vehicles equipped with a GMSL camera (manufactured by SEKONIX, Dongducheon-si, Gyeonggi-do, South Korea). The camera was located above the windshield. There were two cameras with FOVs of $60^{\circ }$ and $120^{\circ }$. In this study, the intrinsic parameters and image undistortion of the camera with $60^{\circ }$ were preconfigured fully. Therefore, a camera with a FOV of $60^{\circ }$ was used to verify this experiment (see Figure 10). This study used linear lane lines that exist at a short distance from the camera (within about 20 m), such as cornerstones or obstacles along the outer edge of the road.

### 4.2. Results

For each scenario, the table provides the average error (avgE), minimum error (minE), maximum error (maxE), and standard deviation (Stdev) for all the control algorithms. This enabled the assessment of their performance in terms of accuracy and stability.

To ensure the validity and efficiency of the proposed method, in the system design, a KF is added at the end of the VP and angle estimation process to filter out false VP detection or miscalculations. The performance of [14] consistently exhibits larger errors than the method proposed by [15]; therefore, its results are often not visible in the graphs.

**Figure 11:**
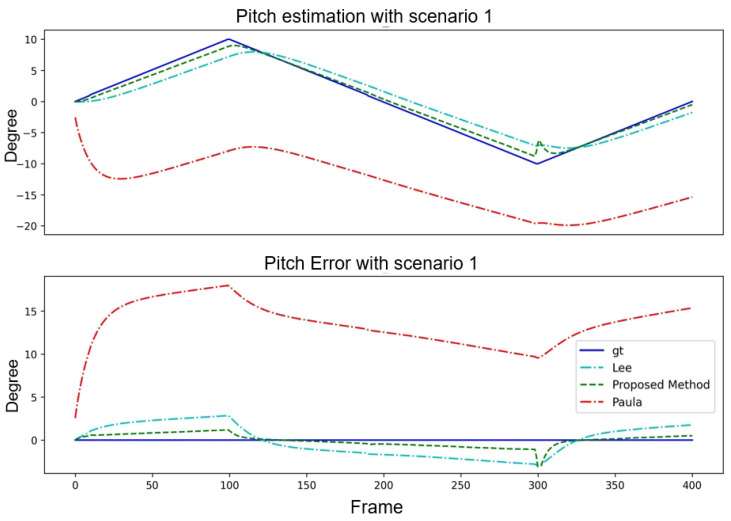
Performance comparison of three algorithms with Scenario 1.

Table 2 presents a quantitative analysis of the pitch angle estimation with a fixed yaw angle. As shown in the table, the proposed method outperformed the methods of [14,15] regarding the avgE, minE, maxE, and Stdev. This indicated that the proposed method is more accurate and stable for pitch angle estimation. This is crucial for robotics and computer vision applications. The angle estimation and error curve results are shown in Figure 11.

**Figure 12:**
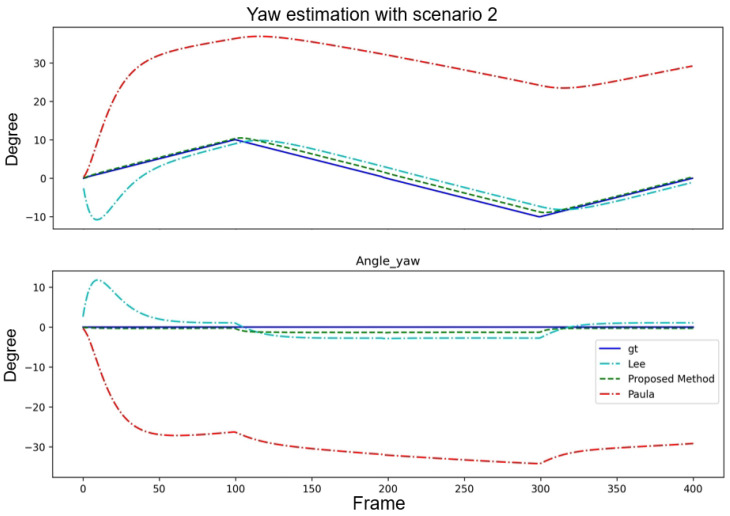
Performance comparison of three algorithms with Scenario 2.

Table 3 presents a quantitative analysis of the yaw angle estimation with a fixed pitch angle. Compared with the method of Paula et al., the proposed method demonstrated superior performance in the minE, maxE, and Stdev. Although the method of [15] performed marginally better in terms of the avgE, the proposed method maintained a balanced performance across all the metrics. This demonstrated its effectiveness for yaw angle estimation. The results of the angle estimation and error curves are shown in Figure 12.

**Figure 13:**
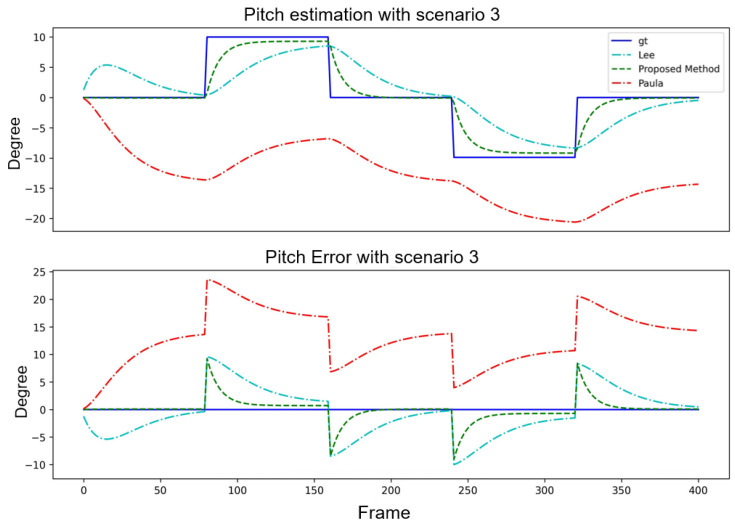
Performance comparison of three algorithms with Scenario 3.

**Figure 14:**
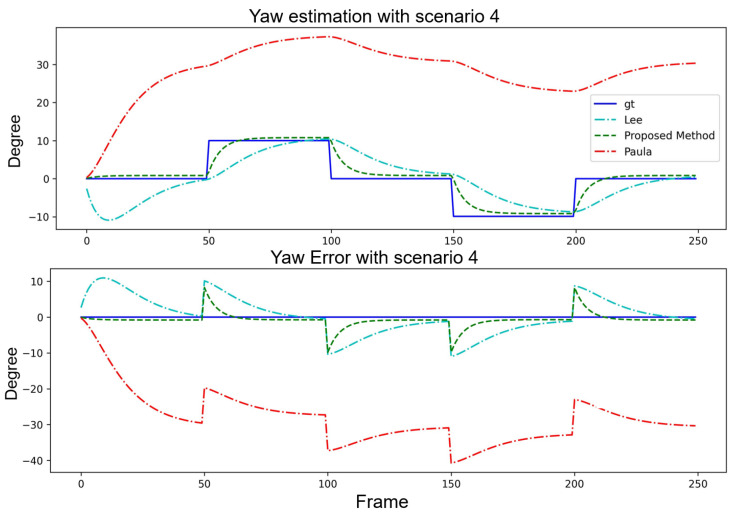
Performance comparison of three algorithms with Scenario 4.

Table 4 and Table 5 focus on the step scenarios with pitch angle estimation under a fixed yaw angle and with yaw angle estimation under a fixed pitch angle. The proposed method exhibited advantages regarding specific error metrics such as the minE and maxE in both cases. Although the proposed method did not outperform [15] in all the categories, its overall performance remained competitive and stable. This further emphasized the robustness and reliability of the proposed method in various scenarios. The system convergence interval was approximately 30 frames. The angles estimation and error curve result are shown in Figure 13 and Figure 14.

**Figure 15:**
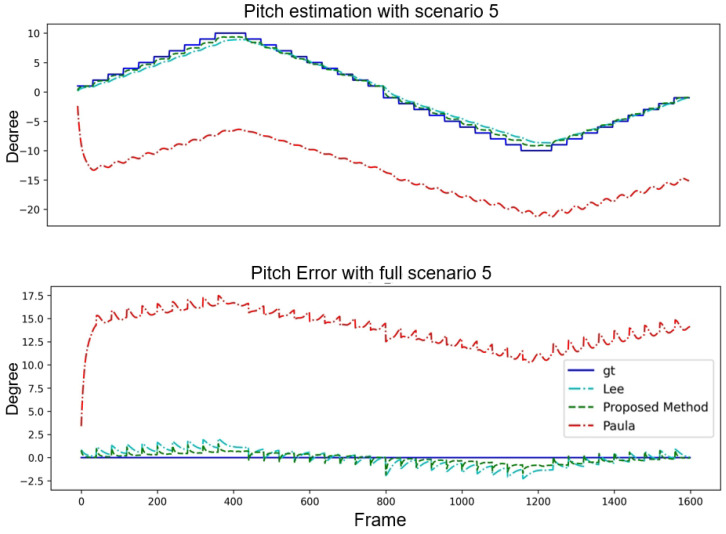
Pitch performance comparison of three algorithms with Scenario 5.

**Figure 16:**
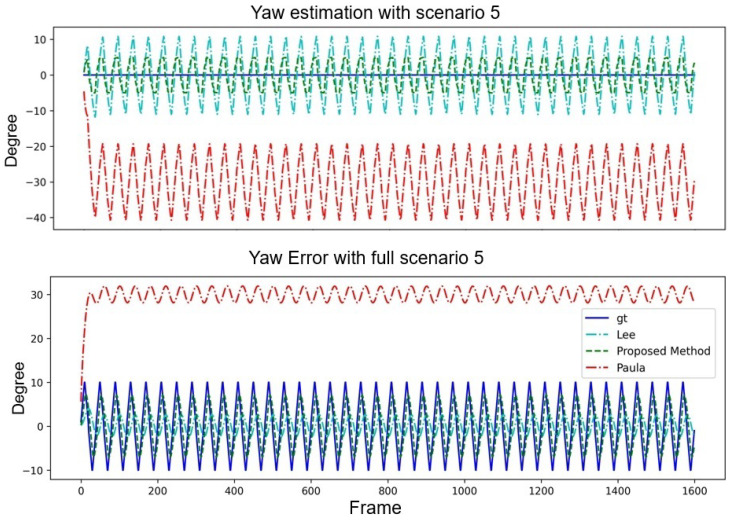
Yaw performance comparison of three algorithms with Scenario 5.

It can be observed from Figure 15 that the proposed method is very close to the lines of the GT and, therefore, has a high accuracy in estimating the pitch angle. The methods in [14,15] seem to have larger errors, especially in some frames, where the errors are pretty significant. In Figure 16, the proposed method again shows characteristics that are very close to the GT, especially in the error plot; the error of the method is relatively small. However, the yaw angle error of the method of [15] is significant in most frames, and although the method of [14] seems to be close to GT in some frames, the error is also large in other frames.

Table 6 presents a quantitative analysis of the orientation using three estimation methods to pitch and yaw for real vehicle datasets. The performances of the methods of [14,15] and that of the proposed method were compared in terms of the avgE, steady-state error (ssE), and Stdev for both pitch and yaw angles.

As observed from the table, the proposed method demonstrates superior performance in terms of the avgE and Stdev for both pitch and yaw angles compared with the other two methods. The proposed method achieved the lowest avgE for pitch and yaw estimation. This indicated its higher accuracy in orientation estimation for real vehicle datasets. Furthermore, the proposed method displayed the lowest Stdev for yaw estimation and a competitive Stdev for pitch estimation. This illustrated the stability and reliability of the method.

## 5. Ablation Study

This study introduces a compensation module based on the foundational mathematical model proposed by [15]. Furthermore, an ablation study was conducted to evaluate the efficacy of various compensation modules. The following methods were analyzed: the original model proposed by [15] (designated as “No process”), Linear Regression (designated as “Linear”), Plane Function Compensation (designated as “Function”), and MLP.

**Figure 17:**
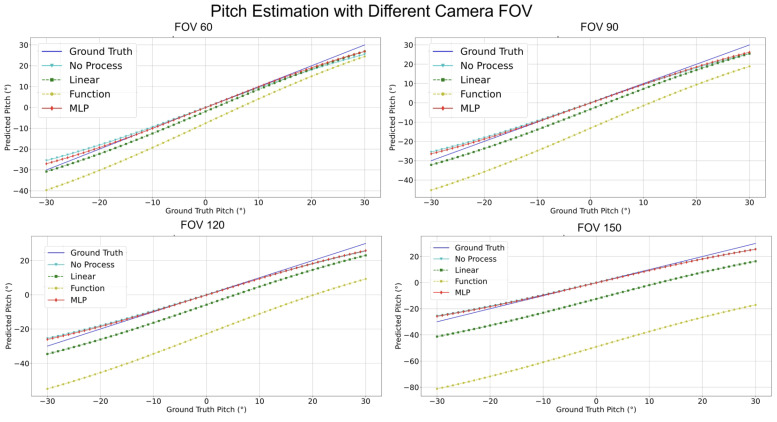
Compensate pitch performance comparison with different camera FOVs.

**Figure 18:**
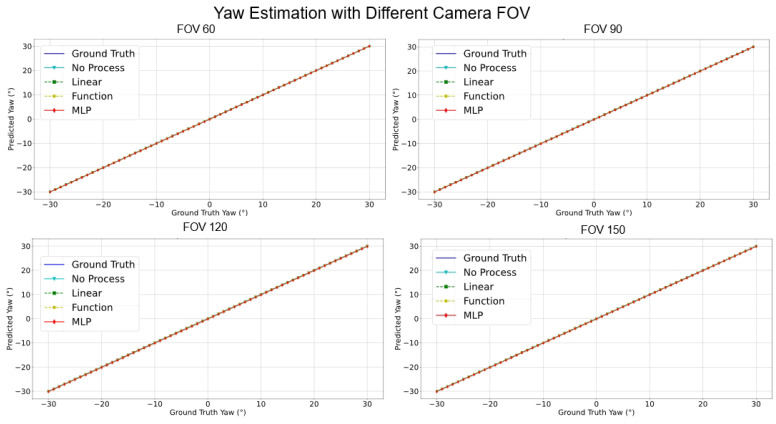
Compensate yaw performance comparison with different camera FOVs.

**Figure 19:**
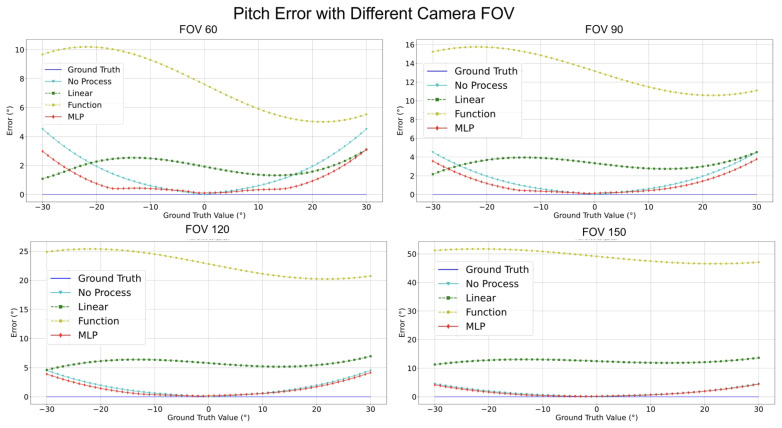
Compensate pitch error comparison with different camera FOVs.

**Figure 20:**
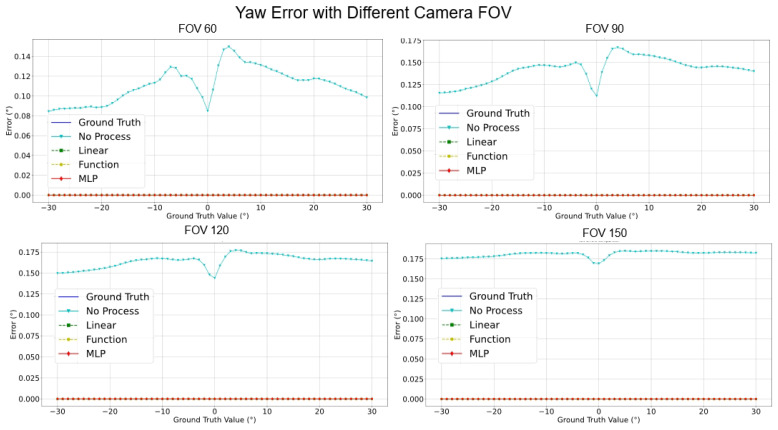
Compensate yaw error comparison with different camera FOVs.

Figure 17, Figure 18, Figure 19 and Figure 20 elucidate the estimation performance of pitch and yaw angles across cameras with different fields-of-view (FOVs), specifically, 60°, 90°, 120°, and 150°. This exploration covers multiple pivotal aspects:

- **Pitch Analysis**: The MLP method consistently outperformed other techniques in estimating pitch angles across all FOV ranges. Relative to GT, the estimations from MLP consistently exhibited remarkable accuracy. Conversely, the “No process” method presented substantial deviations from true values.
- **Yaw Analysis**: Several methods achieved commendable precision for yaw estimations across most FOVs. Nonetheless, the “Linear” encountered marginal error increments at specific angles, while the accuracy of the MLP remained relatively invariant.
- **FOV Assessment**: Across varied FOVs, the MLP method stood out for its pitch and yaw angle estimations accuracy. While the linear and other methods demonstrated efficacy within certain angular ranges, they exhibited noticeable deviations under particular conditions.

Table 7 conveys a comprehensive quantitative evaluation of the ablation studies across different FOVs. This table contrasts the performance metrics of the four methodologies on a simulated dataset. Key insights from this analysis include:

- **Estimation Error for Pitch**: Across all FOVs, MLP consistently registered the most minor error. Notably, at a 150° FOV, its performance superiority was markedly evident. The error of the Linear Regression trajectory steeply ascended with FOV increments. Notably, the error magnitude for the “Function” surged most significantly with FOV enlargement.
- **Estimation Error for Yaw**: Astonishingly, both Linear Regression and Function Compensation methods yielded zero error across all FOVs, epitomizing impeccable estimations. Similarly, the performance of MLP mirrored this perfection. In comparison, the “No process” method, while competent, exhibited a marginal error increase as the FOV expanded.
- **Processing Time Assessment**: The “Function” consistently achieved the swiftest processing times across all FOVs, signifying optimal resource efficiency. In contrast, the “Linear” generally demanded more prolonged processing intervals. MLP and the “No process” methods displayed commendable consistency in processing durations across all FOVs.

While the MLP methodology showcased exemplary precision in pitch and yaw estimations, its consistency in processing durations substantiated its robustness. “Function”, while unparalleled in processing speed, witnessed occasional challenges in estimation accuracy, especially under expanded FOVs. This analysis underscores the intricate balance between speed, resource allocation, and estimation accuracy, suggesting the potential superiority of MLP in wide-ranging practical applications.

## 6. Discussions

### 6.1. Practical Application and Limitations

**Figure 21:**
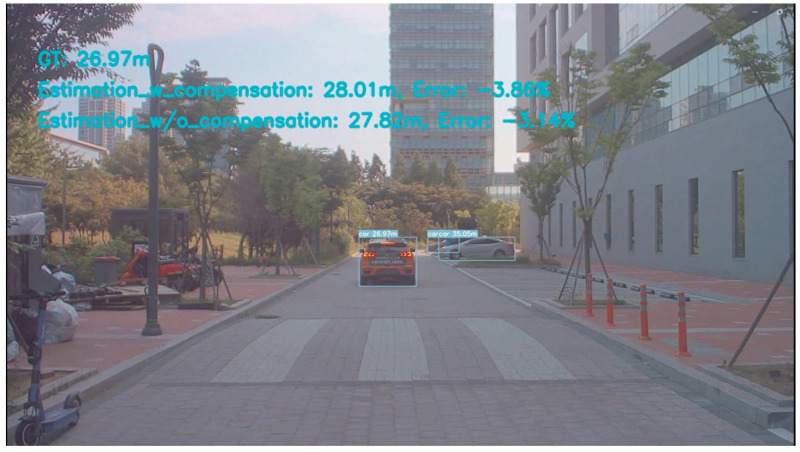
Application sample image of the proposed method.

**Figure 22:**
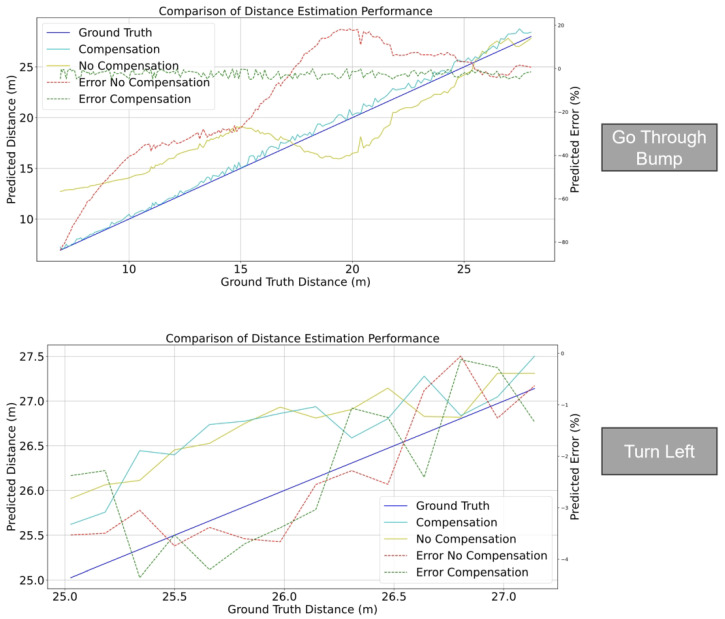
Distance estimation performance comparison between off-line calibration and the proposed method.

The practical application of this study is monocular camera object-based distance estimation. The sample image is shown in Figure 21. There are four scenarios for distance estimation: go through the bump, back through the bump, turn left, and turn right. Here, just discuss the scenario result of going through the bump and turning left as an example as shown in Figure 22. Figure 22 shows two cases of distance estimation with the proposed method. When the vehicle goes through the bump, the compensated one works more than the uncompensated one and the errors of the compensated one are less than 5%. And when the vehicle turns left, the compensated one and uncompensated one are the same: their errors are less than 5%. The total sample videos, MLP weights, and figures of application are shared in the GitHub link https://github.com/leolixingyou/on-the-fly_camera_calibration_MLP_compensation (accessed on 5 December 2023).

**Figure 23:**
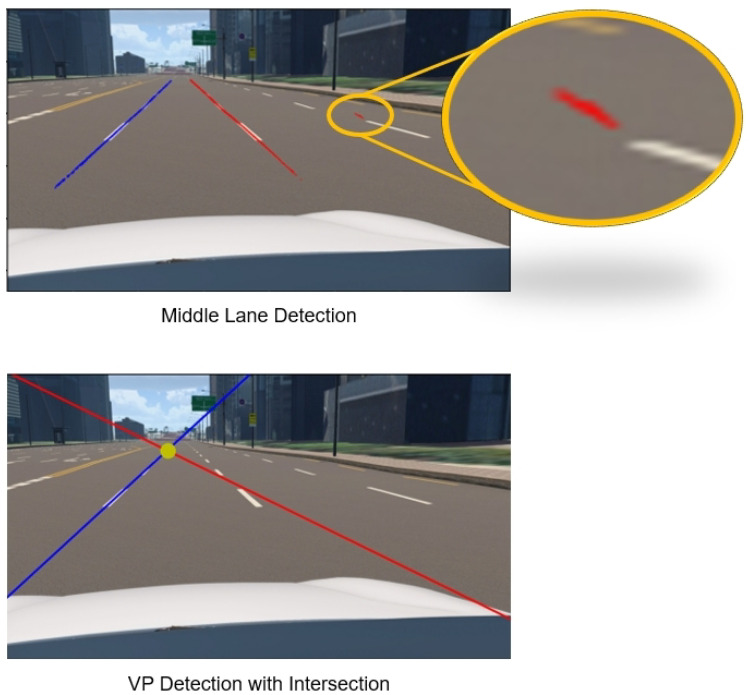
Limitations in terms of VP detection.

Although the compensation part effectually increases the orientation estimation performance, the additional errors happened from different parts. The findings, as represented in Figure 11, show that when the pitch is $-10^{\circ }$ there is a spike. The reason is it has false VP detection is because of lane detection. A similar case is shown in Figure 23. When the camera enters or detects a curve, the above-mentioned error phenomenon will often occur, or its predicted value will fluctuate within a considerable range, so its performance has yet to be determined; when the image is without the lane or any landmark then the outline of the road can be detected so that estimation can work normally. The main idea of this study is to obtain one vanishing point from the target plane with the camera heading direction; this is shown in Figure 21.

### 6.2. Future Work

Considering the accuracy of the proposed method and the content mentioned from Section 6.1, future work will be conducted from the following four aspects:

- **Accurate Lane Detection and Its Process**: For more accurate VP prediction, accurate lane instance segmentation or similar instance classification functions are needed. Building an end-to-end deep neural network from VP detection to orientation estimation may be a good research direction.
- **More Accurate Angle Estimation**: The performance of the compensated angles will show relative advantages compared to other methods. However, as far as the method itself is concerned, there is still room for improvement, such as the prediction performance of pitch in the real environment, the generalization of the compensation part to the camera FOV ability, etc.
- **FOV Assessment**: Across varied FOVs, the MLP method stood out for its pitch and yaw angle estimations accuracy. While the linear and other methods demonstrated efficacy within certain angular ranges, they exhibited noticeable deviations under particular conditions.
- **Curve Case Process**: The proposed method fails when cornering, which is a practical shortcoming. Therefore, defining the necessity and application of orientation estimation during curves is also necessary.

## 7. Conclusions

This study introduces an MLP-based error compensation method for camera orientation estimation using lane lines and a vanishing point. The aim is to identify an algorithm for Automatic On-the-Fly Camera Orientation Estimation and assess its accuracy. An ablation study compared various compensation modules, including Not Processed, Linear Regression, Function, and MLP. The results showed that the MLP compensation improved the original accuracy of the algorithm, especially in estimating pitch and yaw angles. Future research could expand the calibration approach to include the full rotation matrix and camera elevation. With its potential in autonomous vehicles and driver-assistance systems, the method offers promise for real-time camera orientation tasks.

## Figures and Tables

**Table 1:** Comparative analysis of seven simulators.

Simulator	Image Resolution	FOV	Camera Model	Camera Pose	Map Generating	CPU/GPU Minimum Requirements
LGSVL [25]	0∼1920 × 0∼1080	30∼90	Idea	R (0.1)/T (0.1)	-	4 GHz Quad core CPU/GTX 1080 8 GB
Carmaker [26]	0∼1920 × 0∼1920	30∼180	Physical	R (0.1)/T (0.1)	Unity	RAM 4 GB 1 GHz CPU/-
CARLA [27]	0∼1920 × 0∼1920	30∼180	Physical	R (0.1)/T (0.1)	Unity/Unreal	RAM 8 GB Inter i5/GTX 970
NVIDIA DRIVE Sim [28]	0∼1920 × 0∼1920	30∼200	Physical	-	Unity/Unreal	RAM 64 GB/RTX 3090
Isaac Sim [29]	0∼1920 × 0∼1920	0∼90	Physical	R (0.001)/ T (10 × 10^−5^)	Unity/Unreal	RAM 32 GB Inter i7 7th/RTX 2070 8 GB
Air Sim [30]	0∼1920 × 0∼1920	30∼180	Physical	R (0.1)/T (0.1)	Unity/Unreal	RAM 8 GB Inter i5/GTX 970
MORAI [31]	0∼1920 × 0∼1920	30∼179	Idea/Physical	R (10 × 10^−6^)/ T (10 × 10^−6^)	Unity	RAM 16 GB I5 9th/RTX 2060 Super

**Table 2:** Quantitative analysis of pitch estimation with Scenario 1.

	Paula et al. [14]	Lee et al. [15]	Proposed Method
avgE	2.551	0.020	0.015
minE	3.865	0.130	0.120
maxE	4.958	0.236	0.220
Stdev	2.525	1.817	1.352

**Table 3:** Quantitative analysis of yaw estimation with Scenario 2.

	Paula et al. [14]	Lee et al. [15]	Proposed Method
avgE	−29.109	−0.105	−0.566
minE	0.307	0.017	0.002
maxE	34.221	11.810	6.211
Stdev	5.576	3.285	1.726

**Table 4:** Quantitative analysis of pitch estimation with Scenario 3.

	Paula et al. [14]	Lee et al. [15]	Proposed Method
avgE	−10.392	1.465	1.731
minE	−13.210	−0.088	−0.057
maxE	−3.766	3.073	4.317
Stdev	5.013	5.051	5.009

**Table 5:** Quantitative analysis of yaw estimation with Scenario 4.

	Paula et al. [14]	Lee et al. [15]	Proposed Method
avgE	−20.334	4.523	4.804
minE	0.307	0.673	0.112
maxE	38.270	20.763	14.877
Stdev	13.340	9.049	6.347

**Table 6:** Quantitative analysis of orientation using three different pitch and yaw estimations for real vehicle datasets.

Metric	Paula et al. [14]		Lee et al. [15]		Proposed Method	
	**Pitch**	**Yaw**	**Pitch**	**Yaw**	**Pitch**	**Yaw**
avgE	9.241	−22.823	−2.573	−0.297	−2.567	−0.256
ssE	−14.266	24.854	−0.836	−1.400	−0.847	−1.395
Stdev	2.680	2.630	0.165	0.199	0.172	0.156

**Table 7:** Computational cost analysis of each algorithm.

FOV	Accuracy	Method			
		**No Process**	**Linear**	**Function**	**MLP**
60	Pitch/degree	1.572	1.944	7.606	0.881
	yaw/degree	0.111	0	0	0
	Processing time/ms	0.025	0.098	0.003	0.063
	FLOPs	70	80	120	37,318
90	Pitch/degree	1.572	3.344	13.174	1.155
	yaw/degree	0.142	0	0	0
	Processing time/ms	0.025	0.097	0.003	0.063
	FLOPs	70	80	120	37,318
120	Pitch/degree	1.572	5.793	22.817	1.336
	yaw/degree	0.164	0	0	0
	Processing time/ms	0.024	0.094	0.003	0.061
	FLOPs	70	80	120	37,318
150	Pitch/degree	1.572	12.482	49.164	1.473
	yaw/degree	0.181	0	0	0
	Processing time/ms	0.024	0.096	0.003	0.062
	FLOPs	70	80	120	37,318

## Data Availability

The data presented in this study are available on request from the corresponding author. The data are not publicly available due to ongoing validations and continuous improvements.
